# An Active Catalyst System Based on Pd (0) and a Phosphine-Based Bulky Ligand for the Synthesis of Thiophene-Containing Conjugated Polymers

**DOI:** 10.3389/fchem.2021.743091

**Published:** 2021-09-07

**Authors:** Meifang Liu, Li Liu, Zhihui Zhang, Meixiu Wan, Huanmei Guo, Dan Li

**Affiliations:** ^1^ Department of Chemistry and Chemical Engineering, Weifang University, Weifang, China; ^2^ Department of Continuing Education, Weifang Nursing Vocational College, Weifang, China; ^3^ Institute of New Energy Technology, College of Information Science and Technology, Jinan University, Guangzhou, China

**Keywords:** cross-coupling, thiophene boronic acid pinacol ester, aryl halides, polymerization, hyperbranched polymers

## Abstract

To address the limitations of conventional Pd catalysts in the polymerization of thiophene-containing conjugated polymers, an active catalyst system based on Pd (0) and a phosphine-based bulky ligand, L1, is explored systematically in Suzuki–Miyaura polymerizations using thiophene boronic acid pinacol ester as one of the monomers. This active catalyst is found very efficient in synthesizing a series of thiophene-containing linear and hyperbranched conjugated polymers. First, as a model example, coupling reactions between electron-rich/moderately hindered aryl or thienyl halides and thiophene boronic acid pinacol ester give excellent yields with lower catalyst loading and can be completed in a shorter reaction time relative to Pd(PPh_3_)_4_. Notably, high molecular weight thiophene-containing polymers are successfully synthesized by Suzuki–Miyaura polycondensation of 2,5-thiophene bis(boronic acid) derivatives with different dibromo- and triple bromo-substituted aromatics in 5–15 min.

## Introduction

π-Conjugated polymers have received considerable interest for their potential in a variety of applications, such as optoelectronics, chemical sensors, and biological sensors (Ponder et al., 2018; Yue et al., 2019; Li and Pu, 2019; González et al., 2019; Ochieng, et al., 2020; Abdollahi and Zhao, 2020). In particular, conjugated polymers containing thiophene, tri-phenylamine, and benzo(lmn)(3,8)phenanthroline-1,3,6,8(2H,7H)-tetraone in the main or side chains have attracted much attention due to their unique optophysical properties, and they can be used as active components for light-emissive and charge carrier thin-film materials (Koyuncu, 2012; Ma et al., 2013; Ponder et al., 2018; Wu et al., 2018; Li and Pu, 2019; Jessop et al., 2020). Linear conjugated polymers usually have a rigid structure and are easy to form aggregation or crystallization in solvent and solid films. The aggregation of conjugated polymer chains in the film can greatly reduce the luminescence quantum efficiency and is detrimental to the device performance in light-emitting applications. Therefore, in recent years, hyperbranched conjugated polymers with a three-dimensional structure have attracted extensive interest. They mostly possess good solubility, processability, and adjustable photophysical and chemical properties, with effectively inhibited aggregation in the solid state (Xia et al., 2007; Okamoto et al., 2013; Wu et al., 2015; Jiang et al., 2019; Yen and Liou, 2019).

The palladium-catalyzed Suzuki–Miyaura cross-coupling is a mild and efficient reaction to construct the carbon–carbon bonds among aromatics (Bellina et al., 2004; Han, 2013; Paul et al., 2015; Qiu et al., 2018; Rizwan et al., 2018; Ayogu and Onoabedje, 2019; Zhang et al., 2019). This reaction is advantageous over alternative reactions, such as Kumada–Corriu (Clagg et al., 2019; Loewe et al., 1999), Negishi (Chen and Rieke, 1992; Pei et al., 2002; Abdiaj et al., 2018), and Stille (Zou et al., 2009; Rathod et al., 2017), with regard to the tolerance to many kinds of functional groups, the commercial availability of various boronic acids, the nontoxicity and the stability of the catalyst, and the easy separation of by-products (Shen, 1997; Buchwald et al., 1998). Great efforts on the development of catalyst systems for Suzuki–Miyaura cross-coupling reactions have been made over the past 2 decades by Buchwald (Littke and Fu, 2002; Altenhoff et al., 2004; Buchwald et al., 2004; Barder et al., 2005), Beller (Beller et al., 2004), Bedford (Bedford, 2003; Bedford et al., 2003a; Bedford et al., 2003b), Fu (Littke et al., 2000; Liu et al., 2001), Herrmann (Böhm et al., 2000), Norlan (Zhang et al., 1999; Grasa et al., 2002), etc. It should be noted that the catalyst system based on Pd (0) with the electron-rich and bulky phosphorus ligands showed high activity even with existing hindered and electron-rich aryl chloride substrates, and the synthesis of thiophene-containing polymers by Suzuki polymerization is performed successfully by using aryl boronic acids and thiophene halides as starting materials (Littke and Fu, 2002; Altenhoff et al., 2004; Buchwald et al., 2004; Barder et al., 2005; Zou et al., 2009). Because of deboronation of thiophene boronic acid pinacol ester at high temperature, it is difficult to obtain the thiophene-containing products with excellent yields and high molecular weights from electron-rich thiophene boronic acid pinacol ester by Suzuki polymerization. Only few groups reported that high molecular weight polymers were obtained by Suzuki polymerization based on 2,5-thiophenebis (boronic acid pinacol ester)s using Pd(PPh_3_)_4_ as the catalyst precursor (Lu et al., 2008; Liu et al., 2013; Nguyen et al., 2014).

**FIGURE 1:**
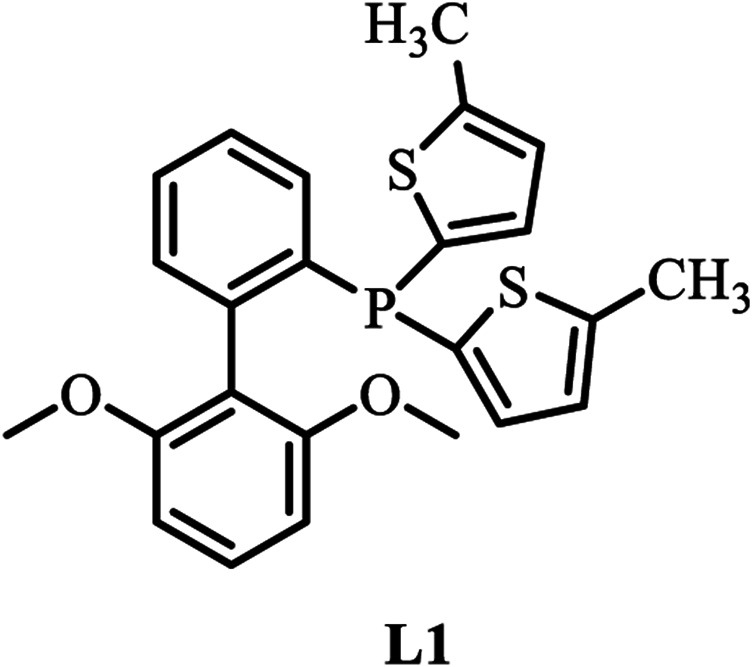
The chemical structure of L1.

A palladium complex containing a bulky electron-rich ligand facilitates the oxidative addition of the aryl halide (Grushin and Alper, 1994; Farina et al., 1997). The chemical structure of L1 is shown in Figure 1. The alkoxy groups attached to the second phenyl ring stabilize the Pd center and prevent cyclometalation, and the thienyl groups on the phosphorus core increase interactions with the Pd center and enhance the electron density of the phosphine-based ligand backbone. These features are beneficial to the activity and lifetime of the catalyst (Ryabov, 1990; Buchwald et al., 2004). The catalyst system consisting of Pd (0) and L1 shows high efficiency for the Suzuki–Miyaura cross-coupling of thiophene-2-boronic ester and aryl halide (Liu et al., 2013). The main drawbacks of the catalytic system Pd (0)/L1 might involve a long reaction time and poor turnover numbers (TONs) and turnover frequencies (TOFs) (Gautam and Bhanage, 2015). Despite the high performance of L1, there is still room to optimize the catalyst system toward a wider scope, higher reactivity, lower catalyst loading, and a shorter reaction time. In this report, L1 was studied as the ligand with zero-valent palladium as the catalyst precursor for Suzuki–Miyaura cross-coupling reaction of benzyl bromide and thiophene boronic acid pinacol ester by changing various reaction conditions including reaction times and the quantities of the catalyst together with the different ratios to ligand L1, and optimized conditions can be obtained. In addition, Suzuki–Miyaura cross-coupling reactions were completed with low levels of catalyst loading and short reaction times for a broad range of substrates. Compared with the traditional palladium source Pd(PPh_3_)_4_, the catalyst Pd_2_(dba)_3_/L1 showed higher performance in generating TON and TOF. Furthermore, this catalyst system can be conducted for Suzuki polycondensation of polymers based on 2,5-thiophenebis (boronic acid pinacol ester), and high molar mass polymers can be easily gained within 15 min.

## Experimental Section

### Materials and Measurements

All chemicals were obtained from commercial suppliers and applied without purification. Solvents were disposed according to the standard process. 5-Bromothiophene-2-carbaldenhyde and L1 were gained according to a previous literature procedure (Li et al., 2008; Liu et al., 2013). The catalyst precursor Pd(PPh_3_)_4_ was prepared according to the literature (Coulson, 1972). All reactions proceeded under N_2_ and monitored by thin-layer chromatography. Column chromatography was conducted on silica gel (200–300 mesh). ^1^H NMR was performed in CDCl_3_ on a Bruker DM 300, AV 400, or AV 600 spectrometer. The gel permeation chromatography (GPC) measurements were performed on a Waters chromatography system connected to a Shimadzu LC-20AD differential refractometer with THF as an eluent or at 150°C with 1,2,4-trichlorobenzene as an eluent and calibration with polystyrene standards.

## Methods

### Pd-catalyzed (Pd_2_(dba)_3_ + L1) Suzuki–Miyaura Coupling of Aryl Bromide or Thienyl Bromide With Thiophene Boronic Ester

A mixture of aryl halides or thienyl halides, thiophene boronic ester, THF (5 L mol^-1^ halide), water, the base (5 equiv), Pd_2_(dba)_3_, and L1 was mixed under nitrogen and refluxed. CH_2_Cl_2_ was then poured into the mixture, and the organic layer was separated and dried with MgSO_4_. The crude product was purified on silica gel eluting with petroleum ether (60–90°C)/acetate ester to provide the title compound.

### Pd-Catalyzed (Pd_2_(dba)_3_ + L1) Suzuki Polycondensation of Aryl Bromide or Thienyl Bromide With Thiophene Boronic Ester

A mixture of aryl halides or thienyl halides, thiophene boronic ester, THF (5 L mol^-1^ halide), water, the base (5 equiv), Pd_2_(dba)_3_, and L1 was mixed under nitrogen and refluxed. Water was then added, and the organic layer was separated and precipitated into methanol. The crude product was purified to provide the polymers.

## Results and Discussion

### Suzuki–Miyaura Cross-Coupling Reaction of Aryl or Thienyl Halide and Thiophene-2-Boronic Acid Pinacol Ester

The Suzuki–Miyaura cross-coupling reactions of thiophene-2-boronic acid pinacol ester and aryl bromide using Pd (0)/L1 have been screened in Table 1. Yields of the isolated product were obtained under various reaction conditions (Table 1). First, decreasing the catalyst loading to 0.1% Pd for reaction of thiophene-2-boronic acid pinacol ester and aryl bromide, the coupling product was obtained in a good yield of 89% after 48 h at 65°C. Interestingly, when the reaction time was shortened to 15 min, there was little effect on the yield of the reaction, and the product was gained in good yields of 85–95% under similar conditions with an increase in the values of TON and TOF (especially by shortening the reaction time to 15 min, the value of TOF increased to 10^3^ h^−1^) (Table 1, entries 3–7). Besides, by further decreasing the Pd loading to 0.01% Pd and using the L1: Pd ratio of 5:1, the process was carried out at 65°C after 0.5 h in yields of 62–89%, and a value of TOF of 2.5–3.6 × 10^4^ h^−1^ could be generated (Table 1, entries 8–12). Among the bases offered in Suzuki–Miyaura cross-coupling reactions, the base K_2_CO_3_ was proved to be the best choice, and the desired products were obtained with the highest yields under the above conditions (Table 1) (Buchwald et al., 2004). According to the results, this catalyst system showed efficient activity for the cross-coupling reactions of aryl bromide and thiophene-2-boronic acid pinacol ester.

**TABLE 1:** Yields of the isolated products from Suzuki–Miyaura cross-coupling reactions of thiophene-2-boronic esters (a) and bromobenzene (b) under various reaction conditions.

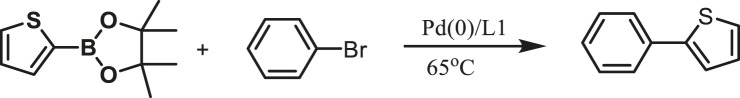							
	Reaction time (h)||base||Catalyst system			a: b	Yield [%]	TON	TOF (h^−1^)
1	48	NaHCO_3_	1%Pd, Pd: L1 = 1:3	1:1	94	94	1.98
2	48	NaHCO_3_	0.1%Pd, Pd: L1 = 1:3	1:1	89	890	18.6
3	0.25	NaHCO_3_	0.1%Pd, Pd: L1 = 1:3	1:1	85	850	3,400
4	0.25	K_2_CO_3_	0.1%Pd, Pd: L1 = 1:3	1:1	95	950	3,800
5	0.25	K_3_PO_4_	0.1%Pd, Pd: L1 = 1:3	1:1	88	880	3,520
6	0.25	Cs_2_CO_3_	0.1%Pd, Pd: L1 = 1:3	1:1	94	940	3,760
7	0.25	Et_3_N	0.1%Pd, Pd: L1 = 1:3	1:1	85	850	3,400
8	0.25	NaHCO_3_	0.01%Pd, Pd: L1 = 1:5	2:1	73	7,300	29,200
9	0.25	K_2_CO_3_	0.01%Pd, Pd: L1 = 1:5	2:1	89	8,900	35,600
10	0.25	K_3_PO_4_	0.01%Pd, Pd: L1 = 1:5	2:1	74	7,400	29,600
11	0.25	Cs_2_CO_3_	0.01%Pd, Pd: L1 = 1:5	2:1	86	8,600	34,400
12	0.25	Et_3_N	0.01%Pd, Pd: L1 = 1:5	2:1	62	6,200	24,800

Reaction conditions: bromobenzene, thiophene-2-boronic acid pinacol ester, 5 equiv. of the base, THF, H_2_O, Pd_2_(dba)_3_ + L1, reflux.

To test the performance of our ligand L1 with low levels of catalyst loading and short reaction times, we chose nine substrates under the conditions of K_2_CO_3_ as the case with 0.1% Pd (0)/L1 (1:3) within 30 min as a test case (Table 2). The isolated yields of the corresponding product are depicted in Table 2. Compared with the catalyst Pd(PPh_3_)_4_, ligand L1 gave better yields in reactions of aryl halide and 2-thiopheneboronic ester under the above conditions. For example, the coupling reaction of bromobenzene or benzyl bromide and aromatic boronic acid pinacol ester with 0.1% Pd (0) (Pd: L1 = 1:3) within 15 min gave excellent isolated yields of the corresponding products, wherein TOFs of 3,840 h^−1^ and 3,800 h^−1^ were obtained, respectively (Table 2, entries 1–2; Supplementary Table S2, entries 1–2). The reaction of electron-rich 1-bromo-4-methoxybenzene and electron-deficient aryl bromide with thiophene-2-boronic acid pinacol ester (ratio 1:1) resulted in the excellent yields of 92–97% with 0.1% Pd (0) (Pd: L1 = 1:3) (Table 2, entries 3–5; Supplementary Table S2, entries 3–5). The above results showed that the presence of electron-rich or electron-deficient groups of aryl bromide had little effect on the yield and TOF of these reactions. In addition, the Suzuki cross-coupling reactions of substrates such as moderately hindered 1,4-dibromo-2,5-dimethylbenzene with thiophene-2-boronic ester could be completed at 0.1% Pd (0) (Pd: L1 = 1:3) to give an 88% yield with a decreased TOF of 880 h^−1^ within 30 min (Table 2, entry 6; Supplementary Table S2, entry 6). Under the similar conditions, Pd(PPh_3_)_4_ furnished the target products with a 24% isolated yield and only a 6 h^−1^ TOF after 2 h. Notably, the Suzuki cross-coupling reactions of 2,5-thiophenebis (boronic ester)s and ortho-substituted bromobenzene Pd (0)/L1 provided the target products in an isolated yield of 90% with a value of TOF of 900 within 30 min, whereas under the similar conditions, Pd(PPh_3_)_4_ gained the isolated yield of 18% with only a 4.5 h^−1^ TOF after 2 h (Table 2, entry 7; Supplementary Table S2, entry 7). Besides, the process worked well on the coupling of electron-rich thienyl bromide and thiophenylboronic ester, and the coupling reactions could be carried out at 0.1% Pd (0) (Pd: L1 = 1:3) to give 90 and 91% yields, with TOFs of 1,800 h^−1^ and 1,820 h^−1^ after 30 min, and Pd(PPh_3_)_4_ gained the isolated yields of 31% and 48%, with TOFs of 15.5 h^−1^ and 24 h^−1^ after 2 h, respectively (Table 2, entries 8–9; Supplementary Table S2, entries 8–9). These results suggest that the catalyst system is remarkably effective for the cross-coupling based thiophene-2-boronic ester with low levels of catalyst loading and short reaction times for a broad range of substrates. Compared with the traditional catalyst precursor Pd(PPh_3_)_4_, the catalyst system Pd_2_(dba)_3_/L1 showed higher performance in yield and TOF. The general mechanism for the Pd-catalytic cross-coupling reaction is divided into three steps, including oxidative addition, transmetalation, and reductive elimination (Miyaura, N. and Suzuki, A. 1995). Oxidative addition is the rate-limiting step for Suzuki–Miyaura coupling reaction, which might be accelerated in the Pd (0)/L1 catalyst system. A high reaction rate can effectively reduce the undesired deboronation of thiophene-2-boronic acid pinacol ester that negatively affects Suzuki–Miyaura cross-coupling reactions under standard conditions (Jayakannan et al., 2001; Kinzel et al., 2010).

**TABLE 2:** Yields of the isolated products from Suzuki–Miyaura cross-coupling reactions of thiophene-2-boronic esters and aryl halides with different catalysts.

	Thiophene-2-boronic esters	Aryl halides	Time (min)	Yield (%)	
				Pd_2_(dba)_3_/L1	Pd(PPh_3_)_4_
1	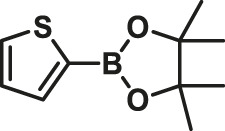	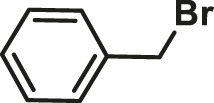	15	96	83
2	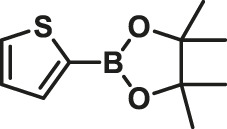	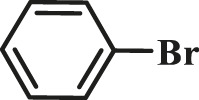	15	95	76
3	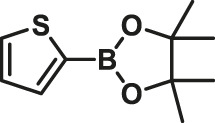	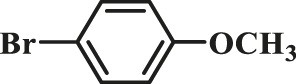	15	92	72
4	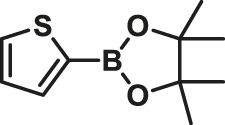	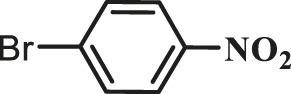	15	93	81
5	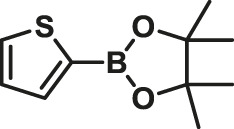	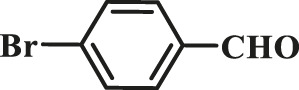	15	97	81
6	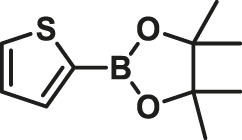	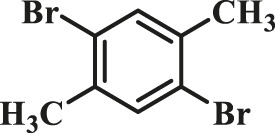	30	88	24
7	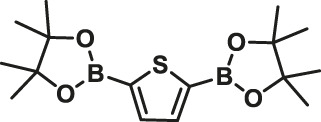	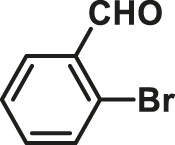	30	90	18
8	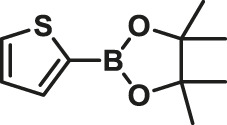	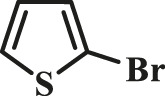	30	90	31
9	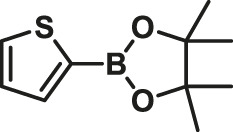	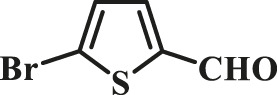	30	91	48

Reaction conditions: 1 equiv. of thienyl halide, 1 or 2 equiv. of thiophenylboronic ester, 5 equiv. of K_2_CO_3_, THF (5 L mol^-1^), H_2_O, 0.1 mol% Pd_2_(dba)_3_ + L1, reflux, within 15–30 min, 1 mol% Pd(PPh_3_)_4_, reflux, within 2 h.

### Suzuki Polycondensation Reaction Based on Thiophenylboronic Ester

**SCHEME 1:**
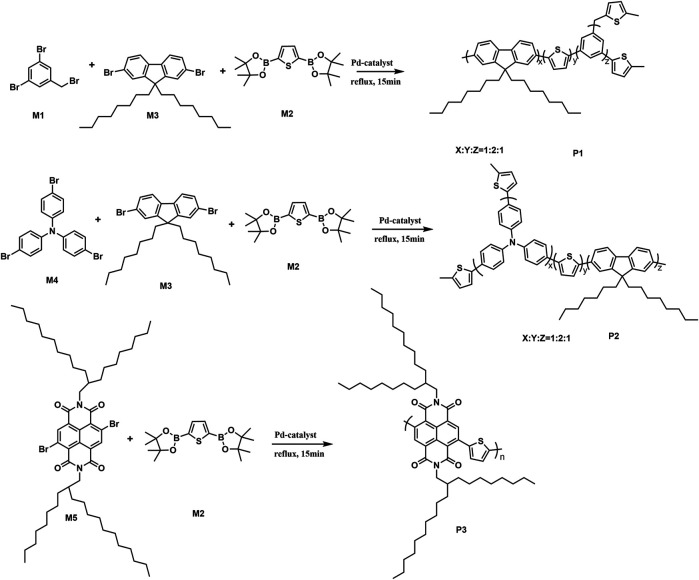
Suzuki polycondensation based on 2,5-thiophenebis (boronic acid pinacol ester) with Pd_2_(dba)_3_ (Pd/L1 = 1/3) and Pd(PPh_3_)_4_ as catalyst precursors.

To test the performance and wide scope of the catalyst precursor Pd (0)/L1, the catalytic system with ligand L1 and Pd_2_(dba)_3_ (Pd/L1 = 1/3) was tested for the synthesis of hyperbranched polymers based 2,5-thiophenebis (boronic acid pinacol ester)s (Scheme 1). For comparison, Pd_2_(dba)_3_ (Pd/L1 = 1/3) and Pd(PPh_3_)_4_ were also applied for the same polymerization as catalyst precursors. Suzuki polycondensation of 2,5-thiophenebis (boronic acid pinacolester)s (M2) with tris(4-bromophenyl)amine (M1) or tris(4-bromophenyl)amine (M4) and 2,7-dibromo-9,9-dioctyl-9H-fluorene (M3) was carried out in a biphasic mixture of THF and aqueous K_2_CO_3_ with freshly prepared Pd_2_(dba)_3_/L1 or Pd(PPh_3_)_4_ as the catalyst precursor in 15 min. The results of polymerization are displayed in Table 3. When using Pd_2_(dba)_3_/L1 as the catalyst precursor, the polycondensation proceeded very rapidly, and large amounts of precipitation were observed in the reaction flask after 5 min. Polymers were not soluble in common reagents such as CHCl_3_ or THF even at the refluxed temperature, but little were soluble in 150°C 1,2,4-trichlorobenzene. The molecule molar masses of P1 and P2 were determined by GPC at 150°C with 1,2,4-trichlorobenzene as the eluent and calibration with polystyrene standards. The soluble fractions of polymers P1 and P2 had molecular weights M*w* of 24,800 g mol^−1^ and 7,900 g mol^−1^, respectively. Using the representative palladium catalyst Pd(PPh_3_)_4_, Suzuki polycondensation of monomers (M1 or M4) and monomer M3 with 2,5-thiophenebis (boronic acid pinacolester)s (M2) provided only low molecule molar mass oligomers with an M*w* less than 3,000 g mol^−1^.

**TABLE 3:** Catalyst precursor, yield, weight-average molecular weight (*M*w), and polydispersity index (PDI).

Polymers	Catalyst precursor	Yield (%)	*M*w^a^(g mol^−1^)	PD
**P1**	Pd_2_(dba)_3_/L1	95	24,800^b^	1.7
**P1**	Pd(PPh_3_)_4_	93	3,000	3.2
**P2**	Pd_2_(dba)_3_/L1	97	7,900^b^	1.4
**P2**	Pd(PPh_3_)_4_	89	2000	1.5
**P3**	Pd_2_(dba)_3_/L1	92	112,000	3.5
**P3**	Pd(PPh_3_)_4_	82	8,400	1.1

a Molecular weight determined by GPC with THF as the eluent, calibrated with polystyrene standards.

b Molecular weight determined by GPC at 150°C with 1,2,4-trichlorobenzene as the eluent, calibrated with polystyrene standards.

**FIGURE 2:**
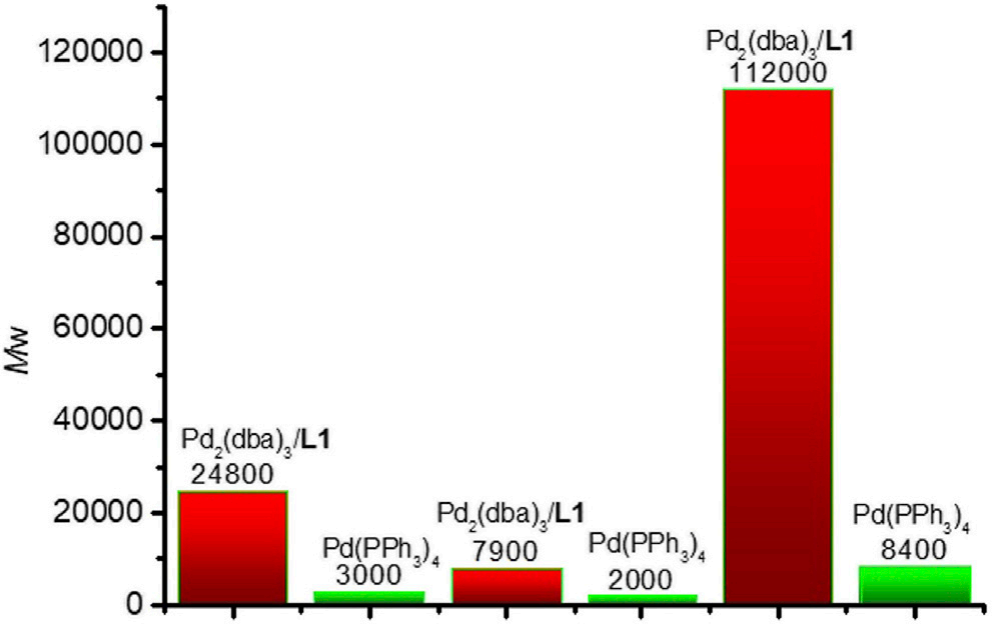
The molecular weight Mw of P1, P2 and P3 with different catalysts.

To test the performance and obtain fully soluble polymers in the reaction solvents, we chose 4,9-dibromo-2,7-bis(2-octyldodecyl)benzo(lmn)(3,8)phenanthroline-1,3,6,8(2H, 7H)-tetraone (M5) and 2,5-thiophenebis (boronic acid pinacolester)s (M2) as monomers. Using Pd_2_(dba)_3_/L1 as the catalyst precursor, polymer P3 was synthesized, which was completely soluble in THF at room temperature in 15 min. The GPC elution curve of P3 showed narrow molecular weight distribution. The number-average molecular weight and weight-average molecular weight of P3 were 32,000 g mol^−1^ and 11,2000 g mol^−1^, respectively (Figure 2). The polycondensation of M2 and M5 was also tested using Pd(PPh_3_)_4_ as the catalyst precursor. GPC data showed that polymer P3 was gained with a molecular weight M*w* 8,400 g mol^−1^. The results are also displayed in Table 3.

## Conclusions

In conclusion, the catalyst system based on Pd (0)/L1 was studied for the Suzuki–Miyaura cross-coupling reactions of thiophene-2-boronic ester with aryl bromides and unactivated thienyl bromides. The catalytic system is efficient in good to excellent yields and high TOFs with low catalyst loadings or shorter reaction times. In addition, relative to Pd(PPh_3_)_4_, this catalyst system also demonstrates higher activity in the Suzuki polymerization of aryl halide and 2,5-thiophenebis (boronic acid pinacol ester)s, resulting in various thiophene-containing conjugated polymers with good yields and high molecular weights within 15 min.

## Data Availability

The original contributions presented in the study are included in the article/Supplementary Material, and further inquiries can be directed to the corresponding authors.
