# Supplementary material for: An Active Catalyst System Based on Pd (0) and a Phosphine-Based Bulky Ligand for the Synthesis of Thiophene-Containing Conjugated Polymers
Source: Front Chem. 2021 Sep 7;9:743091. doi: 10.3389/fchem.2021.743091 (PMC8452923; doi:10.3389/fchem.2021.743091)
Supplement: Supplementary file 1 [file DataSheet1.pdf]

Electronic Supplementary Information (ESI) for:

An Active Catalyst System Based on Pd (0) and Phosphine-based Bulky Ligand for the Synthesis of Thiophene-containing Conjugated Polymers

Meifang Liu[1]*, LiLiu[1], Zhihui Zhang[2], MeiXiu Wan[3], Huanmei Guo[1]*, Dan Li[1]*

[1] Department of Chemistry and Chemical Engineering, Weifang University, Weifang 261061, P. R. China

[2] Department of Continuing Education, Weifang Nursing Vocational College, Weifang 261041, P. R. China

[3] Institute of New Energy Technology, College of Information Science and Technology, Jinan University, Guangzhou 510632, P.R China

* Correspondence:

Meifang Liu, liumf_2011@wfu.edu.cn; Dan Li, danli830109@163.com
Huanmei Guo, huanmeiguo@163.com**

General Procedure [S1]:

Pd-Catalyzed ($Pd_2(dba)_3$ +**L1**) Suzuki-Miyaura Coupling of Aryl Halides or Thienyl Halides with Thiophene boronic Ester

A mixture of aryl halides or thienyl halides, thiophene boronic ester, THF (5 $mLmmol^{-1}$ halide), water, base (5 equiv), $Pd_2(dba)_3$ and **L1** was carefully degassed and charged with nitrogen. The reaction mixture was stirred and refluxed. Ethyl acetate was then added, the organic layer was separated and dried over Na_2SO_4. The crude product was chromatographically purified on silica gel eluting with petroleum ether (60-90 ^oC)/acetate ester to provide the title compound.

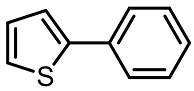

2-phenylthiophene (Table 1) M=160

(1)bromobenzene (M=157, 0.16 g, 1 mmol)**,** 2-thiophenylboronic ester (M=210, 0.21 g, 1 mmol), THF (5 mL), water (1 mL), $NaHCO_3$ (0.42 g, 5 mmol), $Pd_2(dba)_3$ (5.2 mg, 0.01mmol, 1 mol% Pd,) and **L1** (13 mg, 3 mol%), the reaction mixture was stirred and refluxed for 48 h, the title compound was obtained as a white solid (0.15 g, 94%). ^1H NMR (400 MHz, $CDCl_3$) δ: 7.62 (d, 2H), 7.38 (t, 2H), 7.31 (s, 1H), 7.12-7.36 (m, 2H), 7.07 (dd, 1H). The spectrum was in agreement with those described in the literature [S2].

(2)bromobenzene (0.63 g, 4 mmol)**,** 2-thiophenylboronic ester (0.84 g, 4 mmol), THF (20 mL), water (4 mL), $NaHCO_3$ (1.6 g, 20 mmol), $Pd_2(dba)_3$ (2.2 mg, 0.1 mol% Pd) and **L1** (5.3 mg, 0.3 mol%), the reaction mixture was stirred and refluxed for 48 h, the title compound was obtained as a white solid (0.57 g, 89%).

(3)bromobenzene (0.63 g, 4 mmol)**,** 2-thiophenylboronic ester (0.84 g, 4 mmol), THF (20 mL), water (4 mL), $NaHCO_3$ (1.6 g, 20 mmol), $Pd_2(dba)_3$ (2.2 mg, 0.1 mol% Pd) and **L1** (5.3 mg, 0.3

mol%), the reaction mixture was stirred and refluxed for 15 min, the title compound was obtained as a white solid (0.54 g, 85%).

(4)bromobenzene (0.63 g, 4 mmol**,** 2-thiophenylboronic ester (0.84 g, 4 mmol), THF (20 mL), water (4 mL), K_2CO_3 (2.8 g, 20 mmol), $Pd_2(dba)_3$ (2.2 mg, 0.1 mol% Pd) and **L1** (5.3 mg, 0.3 mol%), the reaction mixture was stirred and refluxed for 15 min, the title compound was obtained as a white solid (0.608 g, 95%).

(5)bromobenzene (0.63 g, 4 mmol**,** 2-thiophenylboronic ester (0.84 g, 4 mmol), THF (20 mL), water (4 mL), K_3PO_4 (4.2 g, 20 mmol), $Pd_2(dba)_3$ (2.2 mg, 0.1 mol% Pd) and **L1** (5.3 mg, 0.3 mol%), the reaction mixture was stirred and refluxed for 15 min, the title compound was obtained as a white solid (0.56 g, 88%).

(6)bromobenzene (0.63 g, 4 mmol**,** 2-thiophenylboronic ester (0.84 g, 4 mmol), THF (20 mL), water (4 mL), Cs_2CO_3 (6.5 g, 20 mmol), $Pd_2(dba)_3$ (2.2 mg, 0.1 mol% Pd) and **L1** (5.3 mg, 0.3 mol%), the reaction mixture was stirred and refluxed for 15 min, the title compound was obtained as a white solid (0.60 g, 94%).

(7)bromobenzene (0.78g, 5 mmol), 2-thiophenylboronic ester (2.1 g, 10 mmol, 2 equiv), THF (25 mL), water (5 mL), Et_3N (2.5 g, 25 mmol), $Pd_2(dba)_3$ (0.28 mg, 0.01 mol% Pd) and **L1** (1.1 mg, 0.05 mol%), the reaction mixture was stirred and refluxed for 15 min, the title compound was obtained as a white solid (0.68 g, 85%).

(8)bromobenzene (1.56g, 10 mmol), 2-thiophenylboronic ester (4.2 g, 20 mmol, 2 equiv), THF (50 mL), water (10 mL), $NaHCO_3$ (1.68 g, 20 mmol), $Pd_2(dba)_3$ (0.56 mg, 0.01 mol% Pd) and **L1** (2.2 mg, 0.05 mol%), the reaction mixture was stirred and refluxed for 15 min, the title compound was obtained as a white solid (1.2 g, 73%).

(9)bromobenzene (1.56g, 10 mmol), 2-thiophenylboronic ester (2.1 g, 10 mmol, 2 equiv), THF (50 mL), water (10 mL), K_2CO_3 (6.9 g, 50 mmol), $Pd_2(dba)_3$ (0.56 mg, 0.01 mol% Pd) and **L1** (2.2 mg, 0.05 mol%), the reaction mixture was stirred and refluxed for 15 min, the title compound was obtained as a white solid (1.42 g, 89%).

(10)bromobenzene (1.56g, 10 mmol), 2-thiophenylboronic ester (2.1 g, 10 mmol, 2 equiv), THF (50 mL), water (10 mL), K_3PO_4 (10 g, 50 mmol), $Pd_2(dba)_3$ (0.56 mg, 0.01 mol% Pd) and **L1** (2.2 mg, 0.05 mol%), the reaction mixture was stirred and refluxed for 15 min, the title compound was obtained as a white solid (1.21 g, 74%).

(11)bromobenzene (1.56g, 10 mmol), 2-thiophenylboronic ester (2.1 g, 10 mmol, 2 equiv), THF (50 mL), water (10 mL), Cs_2CO_3 (15 g, 50 mmol), $Pd_2(dba)_3$ (0.56 mg, 0.01 mol% Pd) and **L1** (2.2 mg, 0.05 mol%), the reaction mixture was stirred and refluxed for 15 min, the title compound was obtained as a white solid (1.37g, 86%).

(12)bromobenzene (1.56g, 10 mmol), 2-thiophenylboronic ester (2.1 g, 10 mmol, 2 equiv), THF (25 mL), water (5 mL), NEt_3 (6.9 g, 50 mmol), $Pd_2(dba)_3$ (0.56 mg, 0.01 mol% Pd) and **L1** (2.2 mg, 0.05 mol%), the reaction mixture was stirred and refluxed for 15 min, the title compound was obtained as a white solid (0.99 g, 62%).

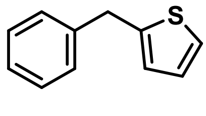

2-benzylthiophene(M=174)

(1)1-bromo-4-methoxybenzene (M=171, 1.71 g, 10 mmol), 2-thiophenylboronic ester (2.1 g, 10 mmol), THF (50 mL), water (10 mL), K_2CO_3 (6.9 g, 50 mmol), $Pd_2(dba)_3$ (5.2mg, 0.1mol % Pd) and L1 (13 mg, 0.3 mol%) were used, the reaction mixture was stirred and refluxed for 15 min, the

product (1.67 g, 96%) was obtained as colorless oil. ^{1}H NMR (600 MHz, $CDCl_3$) δ:7.27-7.31(m, 2H), 7.19-7.24(m, 3H), 7.11(d,1H), 6.90(tr, 1H), 6.78(s, 1H), 4.14(s, 2H). ^{13}C NMR (125 MHz, $CDCl_3$) δ: 144.09, 140.46, 128.65, 128.60, 126.86, 126.54, 125.20, 123.98, 36.10.

(2) 1-bromo-4-methoxybenzene (M=171, 0.171 g, 1 mmol), 2-thiophenylboronic ester (0.21 g, 1 mmol, 1 equiv), THF (5 mL), water (1 mL), K_2CO_3 (0.69 g, 5 mmol), $Pd(PPh_3)_4$ (11 mg, 1 mol % Pd) were used, the reaction mixture was stirred and refluxed for 2 h, the product (0.144 g, 83%) was obtained as colorless oil.

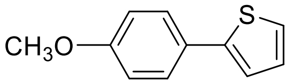

2-(4-methoxyphenyl)thiophene, M=190

(1)1-bromo-4-methoxybenzene (M=187, 1.31 g, 7 mmol)**,** 2-thiophenylboronic ester (1.5 g, 7 mmol), THF (35 mL), water (7 mL), K_2CO_3 (6.9 g, 50 mmol), $Pd_2(dba)_3$ (3.8 mg, 0.1 mol% Pd) and **L1** (9.2 mg, 0.3 mol%), the reaction mixture was stirred and refluxed for 15 min, the product (1.22 g, 92%) was obtained as a white solid. ^{1}H NMR (400 MHz, $CDCl_3$) δ: 8.45-8.10 (dd, 2H), 8.04-7.60 (dd, 2H), 7.60-7.19 (d, 2H), 7.14 (d, 1H). ^{13}C NMR (100 MHz, $CDCl_3$) δ: 159.1,127.9, 127.3, 127.2, 123.8, 122.1, 114.2, 55.3. The spectra were in agreement with those described in the literature [S2].

(2) 1-bromo-4-methoxybenzene (M=187, 0.187 g, 1 mmol)**,** 2-thiophenylboronic ester (0.21 g, 1 mmol, 1 equiv), THF (5 mL), water (1 mL), K_2CO_3 (0.69 g, 5 mmol), $Pd(PPh_3)_4$ (11 mg, 1 mol % Pd) were used, the reaction mixture was stirred and refluxed for 2h, the product (0.137 g, 72%) was obtained as a white solid.

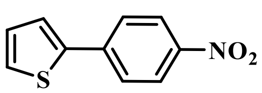

2-(4-nitrophenyl)thiophene M=205

(1)1-bromo-4-nitrobenzene(M=202, 2.02 g, 10 mol), 2-thiophenylboronic ester (2.1 g, 10 mmol), THF (50 mL), water (10 mL), K_2CO_3 (6.9 g, 50 mmol), $Pd_2(dba)_3$ (5.2mg, 0.1mol % Pd) and **L1** (13 mg, 0.3 mol%) were used, the reaction mixture was stirred and refluxed, the product (1.9 g, 93%) was obtained as a solid. ^1H NMR (400 MHz, $CDCl_3$) δ 8.45-8.10 (dd, 2H), 8.04-7.60 (dd, 2H), 7.60-7.19 (m, 2H), 7.14-7.15 (m,1H). ^{13}C NMR (151 MHz, $CDCl_3$) δ: 146.72, 141.69, 140.68, 140.66, 140.65, 128.78, 127.77, 126.11, 125.79, 124.50, 77.34, 77.12, 76.91.

(2) 1-bromo-4-nitrobenzene(M=202, 0.2 g, 1 mol), 2-thiophenylboronic ester (0.21 g, 1 mmol), THF (5 mL), water (1 mL), K_2CO_3 (0.69 g, 5 mmol), $Pd(PPh_3)_4$ (11 mg, 1 mol % Pd)were used, the reaction mixture was stirred and refluxed, the product (0.160g, 78%) was obtained as a solid.

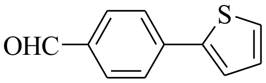

4-(thiophene-2-yl)benzaldehyde, M=188

(1) 4-bromobenzaldehyde (M =185, 1.3 g, 7mmol), 2-thiophenylboronic ester (1.5 g, 7 mmol), THF (35 mL), water (7 mL), K_2CO_3 (3.4 g, 35 mmol), $Pd_2(dba)_3$ (3.8mg, 0.1mol % Pd) and **L1** (9.2 mg, 0.3 mol%) were used, the reaction mixture was stirred and refluxed for 15 min, the product (1.27g, 97%) was obtained as a solid. ^1H NMR (400 MHz, $CDCl_3$) δ: 9.99 (s, 1H), 7.89 (d, 2H), 7.77 (d, 2H), 7.47 (dd, 1H), 7.39 (dd, 1H), 7.11(dd, 1H); The spectrum was in agreement with those described in the literature[S2].

(2) 4-bromobenzaldehyde (0.185 g, 1 mmol), 2-thiophenylboronic ester (0.21 g, 1 mmol, 1 equiv), THF (5 mL), water (1 mL), K_2CO_3 (0.69 g, 5 mmol), $Pd(PPh_3)_4$ (11 mg, 1 mol % Pd)were used, the reaction mixture was stirred and refluxed for 2h, the product (0.15g, 81%) was obtained as a solid.

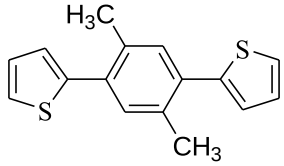

2-(2,5-dimethyl-4-(thiophene-2-yl)phenyl)thiophene, M=270

1,4-dibromo-2,5-dimethylbenzene (M= 264, 1.3 g, 5 mmol)**,** 2-thiophenylboronic ester (2.1 g, 10 mmol, 2 equiv), THF (25 mL), water (5 mL), K_2CO_3 (6.9 g, 50 mmol), $Pd_2(dba)_3$ (5.2 mg, 0.1 mol% Pd) and **L1** (13 mg, 0.3 mol%) were used, the reaction mixture was stirred and refluxed for 30 min. the product (1.18 g, 88%) was obtained as a white solid. ^1H NMR (400 MHz, $CDCl_3$) δ: 7.34-7.36 (m, 4H), 7.10-7.12 (m, 4H), 2.43(s, 6H); ^{13}C NMR (100 MHz, $CDCl_3$) δ: 142.7, 133.5, 133.4, 132.7, 127.1, 126.4, 125.2, 20.6. The spectra were in agreement with those described in the literature[S2].

1,4-dibromo-2,5-dimethylbenzene (M= 264, 0.26 g, 1 mmol), 2-thiophenylboronic ester (0.42 g, 2 mmol, 2 equiv), THF (10 mL), water (2 mL), K_2CO_3 (1.38g, 10 mmol), $Pd(PPh_3)_4$ (22 mg, 1 mol % Pd) were used, the reaction mixture was stirred and refluxed for 2h. the product (0.065 g, 24%) was obtained as a white solid.

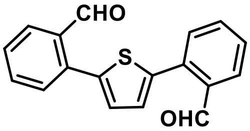

2,2'-(thiophene-2,5-diyl)dibenzaldehyde, M=292

(1) 2-bromobenzaldehyde (M =185, 1.85 g, 10mmol，2 equiv), 2,5-thiophenebis(boronic ester)s (M=336, 1.68 g, 5 mmol, 1 equiv), THF (50 mL), water (10 mL), K_2CO_3 (6.9 g, 50mmol), $Pd_2(dba)_3$ (5.2mg, 0.1mol % Pd) and **L1** (13 mg, 0.3 mol%) were used, the reaction mixture was stirred and refluxed for 30 min, the product (2.6g, 90%) was obtained as a solid. ^1H NMR (600 MHz, $CDCl_3$) δ:10.29 (s, 2H), 8.03 (dd, 2H), 7.66-7.51 (m, 6H), 7.10 (d, 2H).^{13}C NMR (151 MHz, $CDCl_3$) δ 191.64, 140.92, 140.91, 140.89, 137.28, 134.24, 134.23, 133.84, 131.36, 130.53, 130.26, 129.93, 129.67, 129.65, 128.77, 128.25, 128.23.

(2) 2-bromobenzaldehyde (0.37 g, 2 mmol), 2,5-thiophenebis(boronic ester)s (M=336, 0.336 g, 1 mmol, 1 equiv), THF (10 mL), water (2 mL), K_2CO_3 (1.38 g, 10 mmol), $Pd(PPh_3)_4$ (22 mg, 1 mol % Pd)were used, the reaction mixture was stirred and refluxed for 2h, the product (0.052g, 18%) was obtained as a solid.

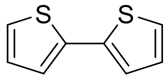

2,2'-bithiophene (Table 3, Entry 1) M=166

2-bromothiophene (M=163, 0.82, 5 mmol, 1.0 equiv), 2-thiophenylboronic ester (1.05 g, 5 mmol, 1 equiv), THF (25 mL), water (5 mL), K_2CO_3 (3.4 g, 25 mmol), $Pd_2(dba)_3$ (2.7 mg, 0.1 mol % Pd) and **L1** (6.5 mg, 0.3 mol%) were used, the mixture was carefully degassed and charged with nitrogen. The reaction mixture was stirred and refluxed for 0.5 h. Ethyl acetate was then added, the organic layer was separated and dried over Na_2SO_4. The crude product was chromatographically purified on silica gel eluting with petroleum ether (60-90 ^oC)/ acetate ester (20/1) to provide the title compound as a green oil the (0.75g, 90%).

2-bromothiophene (M=163, 0.82, 5 mmol, 1.0 equiv), 2-thiophenylboronic ester (1.05 g, 5 mmol, 1 equiv), THF (25 mL), water (5 mL), K_2CO_3 (3.4 g, 25 mmol), $Pd(PPh_3)_4$ (55 mg, 1 mol % Pd) were used, the reaction mixture was stirred and refluxed for 2 h, the product ((0.25 g, 31 %) was obtained.

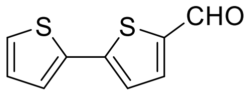

5-(thiophene-2-yl)thiophene-2-carbaldehyde (Table3, Entry 3)M=194

5-bromothiophene-2-carbaldehyde (M=191, 1.91 g, 10 mmol, 1.0 equiv), 2-thiophenylboronic ester (2.1 g, 10 mmol, 1 equiv), THF (50 mL), water (10 mL), K_2CO_3 (4.1 g, 30 mmol), $Pd_2(dba)_3$ (5.2 mg, 0.1 mol % Pd) and **L1** (13 mg, 0.3mol %) (Pd: **L1** = 1: 3) were used, the mixture was carefully

degassed and charged with nitrogen. The reaction mixture was stirred and refluxed for 0.5 h. Et_2O was then added, the organic layer was separated and dried over Na_2SO_4. The crude product was chromatographically purified on silica gel eluting with petroleum ether (60-90 ^oC)/acetate ester (10/1) to provide the title compound the product (1.76 g, 91%) was obtained as a pale yellow solid. ^1H NMR (400 MHz, $CDCl_3$) δ 9.85 (s,1H), 7.65 (d, 1H), 7.35 (dd, 1H), 7.34 (s, 1H), 7.27-7.21 (m, 1H), 7.06 (dd, 1H). The spectrum was in agreement with those described in the literature[S3].

5-bromothiophene-2-carbaldehyde (M=191, 0.191 g, 1 mmol, 1.0 equiv.), 2-thiophenylboronic ester (0.21 g, 1 mmol, 1 equiv), THF (5 mL), water (1 mL), K_2CO_3 (0.69 g, 5 mmol), $Pd(PPh_3)_4$ (11 mg, 1 mol % Pd) were used, the reaction mixture was stirred and refluxed for 2 h, the product ((0.093 g, 48 %) was obtained.

Turnover number refers to the number of reacted molecules per surface active site. (TON)Turnover frequency (TOF) refers to the number of reacted molecules per surface active site per time for a catalytic reaction.[S4]

Table S1.TON and TOF of the isolated products from Suzuki-Miyaura cross-coupling reactions of thiophene-2-boronic esters (a) and bromobenzene (b) under various reaction conditions.

	Time	Base	Catalyst	Yield [%]	TON	Calculation Details (TOF)	TOF(h^{-1})
1	48	$NaHCO_3$	1%Pd, Pd: L1=1:3	94	94	$\frac{0.94 \times 1\times10^{-3}}{1\times10^{-3}\times\frac{1}{100}\times48}$	1.98
2	48	$NaHCO_3$	0.1%Pd, Pd: L1=1:3	89	890	$\frac{0.89 \times 4\times10^{-3}}{4\times10^{-3}\times\frac{0.1}{100}\times48}$	18.6
3	0.25	$NaHCO_3$	0.1%Pd, Pd: L1=1:3	85	850	$\frac{0.85 \times 4\times10^{-3}}{4\times10^{-3}\times\frac{0.1}{100}\times0.25}$	3400

4	0.25	K_2CO_3	0.1%Pd, Pd: L1=1:3	95	950	$\frac{0.95 \times 4 \times 10^{-3}}{4\times10^{-3}\times \frac{0.1}{100} \times 0.25}$	3800
5	0.25	K_3PO_4	0.1%Pd, Pd: L1=1:3	88	880	$\frac{0.88 \times 4 \times 10^{-3}}{4\times10^{-3}\times \frac{0.1}{100} \times 0.25}$	3520
6	0.25	Cs_2CO_3	0.1%Pd, Pd: L1=1:3	94	940	$\frac{0.94 \times 4 \times 10^{-3}}{4\times10^{-3}\times \frac{0.1}{100} \times 0.25}$	3760
7	0.25	Et_3N	0.1%Pd, Pd: L1=1:3	85	850	$\frac{0.85 \times 4 \times 10^{-3}}{4\times10^{-3}\times \frac{0.1}{100} \times 0.25}$	3400
8	0.25	$NaHCO_3$	0.01%Pd, Pd: L1=1:5	73	7300	$\frac{0.73 \times 5 \times 10^{-3}}{5\times10^{-3}\times \frac{0.01}{100} \times 0.25}$	29200
9	0.25	K_2CO_3	0.01%Pd, Pd: L1=1:5	89	8900	$\frac{0.89 \times 10 \times 10^{-3}}{10\times10^{-3}\times \frac{0.01}{100} \times 0.25}$	35600
10	0.25	K_3PO_4	0.01%Pd, Pd: L1=1:5	74	7400	$\frac{0.74 \times 10 \times 10^{-3}}{10\times10^{-3}\times \frac{0.01}{100} \times 0.25}$	29600
11	0.25	Cs_2CO_3	0.01%Pd, Pd: L1=1:5	86	8600	$\frac{0.86 \times 10 \times 10^{-3}}{10\times10^{-3}\times \frac{0.01}{100} \times 0.25}$	34400
12	0.25	Et_3N	0.01%Pd, Pd: L1=1:5	62	6200	$\frac{0.62 \times 10 \times 10^{-3}}{10\times10^{-3}\times \frac{0.01}{100} \times 0.25}$	24800

Table S2. TON, TOF of the isolated products from Suzuki-Miyaura cross-coupling reactions of thiophene-2-boronic esters and aryl halides with different catalysts.

	product	$Pd_2(dba)_3$/**L1**			$Pd(PPh_3)_4$		
		TON	calculation details (TOF)	TOF(h^{-1})	TON	calculation details(TOF)	TOF (h^{-1})
1	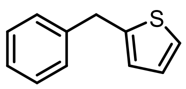	960	$\frac{0.96\times10\times10^{-3}}{10\times10^{-3}\times\frac{0.1}{100}\times0.25}$	3840	83	$\frac{0.83 \times 1 \times10^{-3}}{1\times10^{-3}\times\frac{1}{100} \times 2}$	41.5
2	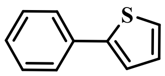	950	$\frac{0.95\times4\times10^{-3}}{4\times10^{-3}\times\frac{0.1}{100}\times0.25}$	3800	76	$\frac{0.76\times1\times10^{-3}}{1\times10^{-3}\times\frac{1}{100}\times2}$	38
3	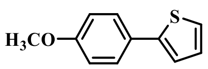	920	$\frac{0.92\times7\times10^{-3}}{7\times10^{-3}\times\frac{0.1}{100}\times0.25}$	3680	72	$\frac{0.72\times1\times10^{-3}}{1\times10^{-3}\times\frac{1}{100}\times2}$	36
4	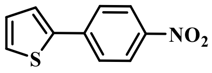	930	$\frac{0.93\times10\times10^{-3}}{10\times10^{-3}\times\frac{0.1}{100}\times0.25}$	3720	78	$\frac{0.78\times1\times10^{-3}}{1\times10^{-3}\times\frac{1}{100}\times2}$	39

5	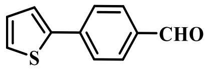	970	$\frac{0.97x7x10^{-3}}{7x10^{-3}x\frac{0.1}{100} x 0.25}$	3880	81	$\frac{0.81x1x10^{-3}}{1x10^{-3}x\frac{1}{100} x2}$	40.5
6	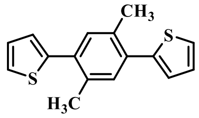	440	$\frac{0.88 x 5 x10^{-3}}{5x10^{-3}x\frac{0.1}{100} x 2x0.5}$	880	12	$\frac{0.24x1x10^{-3}}{1x10^{-3}x\frac{1}{100} x 2x2}$	6
7	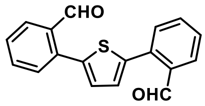	450	$\frac{0.90 x 10 x10^{-3}}{10x10^{-3}x \frac{0.1}{100} x 2x0.5}$	900	9	$\frac{0.18x1x10^{-3}}{1x10^{-3}x\frac{1}{100} x 2x2}$	4.5
8	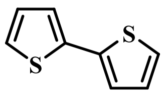	900	$\frac{0.90 x 5 x10^{-3}}{5x10^{-3}x \frac{0.1}{100} x0.5}$	1800	31	$\frac{0.31x5x10^{-3}}{5x10^{-3}x\frac{1}{100} x2}$	15.5
9	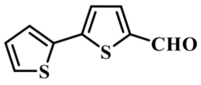	910	$\frac{0.91 x 10 x10^{-3}}{10x10^{-3}x \frac{0.1}{100} x0.5}$	1820	48	$\frac{0.48 x 1x10^{-3}}{1x10^{-3}x\frac{1}{100} x2}$	24

Reaction conditions: 1 equiv. of thienyl halide, 1 or 2 equiv. of thiophenyl-boronic ester, 5 equiv. of K_2CO_3, THF (5 $Lmol^{-1}$), H_2O, 0.1 mol% $Pd_2(dba)_3$+L1, reflux, within 15-30min. 1 mol% $Pd(PPh_3)_4$, reflux, within 2h.

Polymers:

P1: A mixture of 2,5-thiophenebis(boronic acid pinacolester)s (0.5 mmol, 0.17 g), tris(4-bromophenyl)amine (0.2 mmol, 0.065 g) and 2,7-dibromo-9,9-dioctyl-9H-fluorene (0.2 mmol, 0.11 g) fluorene halide, THF (20 ml), water (5 ml), K_2CO_3(0.69 g), $Pd_2(dba)_3$(5 mg) and **L1**(14 mg) was carefully degassed and charged with nitrogen. The reaction mixture was stirred and refluxed for 15 min. Water was then added, polymers were separated by filtration. The crude products were resolved by amount of THF and precipitated into methanol. The former precipitate was collected by filtration to give the polymer **P1** as green solid (0.10 g, 95%). ^{1}H NMR (600 MHz, $CDCl_3$) δ: 8.02-8.03(m, 2H), 7.5-7.65(broad, 9H), 6.89-7.19(m, 1H), 1.02-1.19(m, 65H), 0.73-0.82(m, 26H).

A mixture of 2,5-thiophenebis(boronic acid pinacolester)s (0.5 mmol, 0.17 g), tris(4-bromophenyl)amine (0.2 mmol, 0.065 g) and 2,7-dibromo-9,9-dioctyl-9H-fluorene (0.2 mmol, 0.11g) fluorene halide, THF (20 ml), water (5 ml), K_2CO_3(0.69 g), $Pd(PPh_3)_4$ (11 mg) was carefully degassed and charged with nitrogen. The reaction mixture was stirred and refluxed for 15 min. Water was then added, polymers were separated by filtration. The crude products were resolved by amount of THF and precipitated into methanol. The former precipitate was collected by filtration to give the polymer P1 as green solid (0.098 g, 93%).

P2: A mixture of 2,5-thiophenebis(boronic acid pinacolester)s(0.25 mmol, 0.085 g), tris(4-bromophenyl)amine (0.1 mmol, 0.0482 g), 2,7-dibromo-9,9-dioctyl-9H-fluorene (0.1 mmol, 0.0548 g), THF(20 ml), water (5 ml) , K_2CO_3(0.39 g), $Pd_2(dba)_3$(3 mg) and **L1**(8 mg) was carefully degassed and charged with nitrogen. The reaction mixture was stirred and refluxed for 15 min. Water was then added, polymers were separated by filtration. The crude products were resolved by amount of THF and precipitated into methanol. The former precipitate was collected by filtration to give the polymer **P2** as green solid (0.118g, 97%).

A mixture of 2,5-thiophenebis(boronic acid pinacolester)s(0.25 mmol, 0.085 g), tris(4-bromophenyl)amine (0.1 mmol, 0.0482 g), 2,7-dibromo-9,9-dioctyl-9H-fluorene (0.1 mmol, 0.0548 g), THF(20 ml), water (5 ml) , K_2CO_3(0.69 g), $Pd(PPh_3)_4$ (6 mg)was carefully degassed and charged with nitrogen. The reaction mixture was stirred and refluxed for 15 min. Water was then added, polymers were separated by filtration. The crude products were resolved by amount of THF and precipitated into methanol. The former precipitate was collected by filtration to give the polymer **P2** as green solid (0.108 g, 89%).^{1}H NMR (600 MHz, $CDCl_3$) δ: 8.02(s, 2H), 7.33-7.63 (broad, 7H), 6.89-7.12(broad, 3H), 1.24-0.74 (m, 50H).

P3: A mixture of 4,9-dibromo-2,7-bis(2-octyldodecyl)benzo[lmn][3,8]phenanthroline-1,3,6,8(2H,7H)-tetraone (0.2 mmol, 0.197 g) and 2,5-thiophenebis(boronic ester)s(0.2 mmol, 0.0672 g), THF (20 ml), water (5 ml) , K_2CO_3 (0.38 g), $Pd_2(dba)_3$(2 mg) and **L1**(6 mg) was carefully degassed and charged with nitrogen. The reaction mixture was stirred and refluxed for 15 min. THF was then added, the organic layer was separated and dried over Na_2SO_4.The crude products were resolved by amount of THF and precipitated into methanol. The former precipitate was collected by filtration to give the polymer **P3** as green solid (0.084 g, 92%). ^{1}H NMR (400 MHz, $CDCl_3$) δ: 8.90 (s, 2H), 7.39 (s, 2H), 4.08 (s, 4H), 1.45-0.73 (m, 78H).

A mixture of 4,9-dibromo-2,7-bis(2-octyldodecyl)benzo[lmn][3,8]phenanthroline-1,3,6,8(2H,7H)-tetraone (0.2mmol, 0.197g) and 2,5-thiophenebis(boronic ester)s(0.2mmol, 0.0672 g), THF(20 ml), water (5 ml) , K_2CO_3(0.38 g), $Pd(PPh_3)_4$ (9 mg) was carefully degassed and charged with nitrogen. The reaction mixture was stirred and refluxed for 15min. THF was then added, the organic layer was separated and dried over Na_2SO_4.The crude products were resolved by amount of THF and precipitated into methanol. The former precipitate was collected by filtration to give the polymer

(0.75 g, 82%).

References

S1. Coulson, D.R. (1972). Tetrakis(triphenylphosphine)palladium(0). *Inorg. Synth.* 13, 121-124. https://doi.org/10.1002/9780470132449.ch23

S2. Liu, M.; Chen, Y.; Zhang, C.; Li, C.; Li, W.; Bo, Z. (**2013**). Synthesis of thiophene-containing conjugated polymers from 2, 5-thiophenebis (boronic ester)s by Suzuki polycondensation. *Polym. Chem*. 4, 895-899. https://doi.org/10.1039/C2PY21070C

S3. Li, W.W.; Han Y.; Li B.S.; Liu C.; Bo, Z.S. (2008). Tris[tri(2-thienyl)phosphine]palladium as the Catalyst Precursor for Thiophene-Based Suzuki-Miyaura Cross-coupling and Polycondensation. *J. Polym. Sci., Part A: Polym. Chem*. 46, 4556-4563. https://doi.org/10.1002/pola.22792

S4. Gautam, P. and Bhanage. B.M. (2015). Palladacycle Catalyzed Carbonylative Suzuki–Miyaura Coupling with High Turnover Number and Turnover Frequency. *J. Org. Chem.* 80, 7810-7815

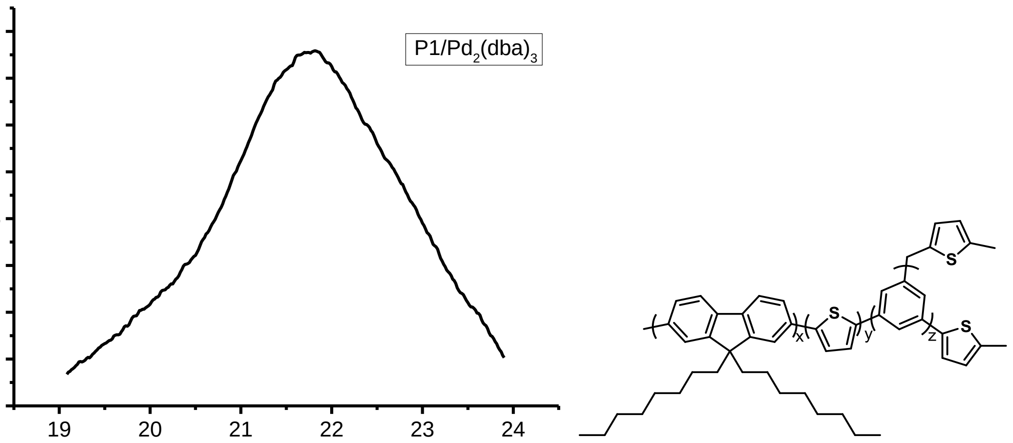

Figure S1. GPC elution curve of polymer **P1** prepared by using $Pd_2(dba)_3$/**L1** as the catalyst precursors. Molecular weight determined by GPC at 150 °C with 1,2,4-trichlorobenzene as eluent, calibrated with polystyrene standards.

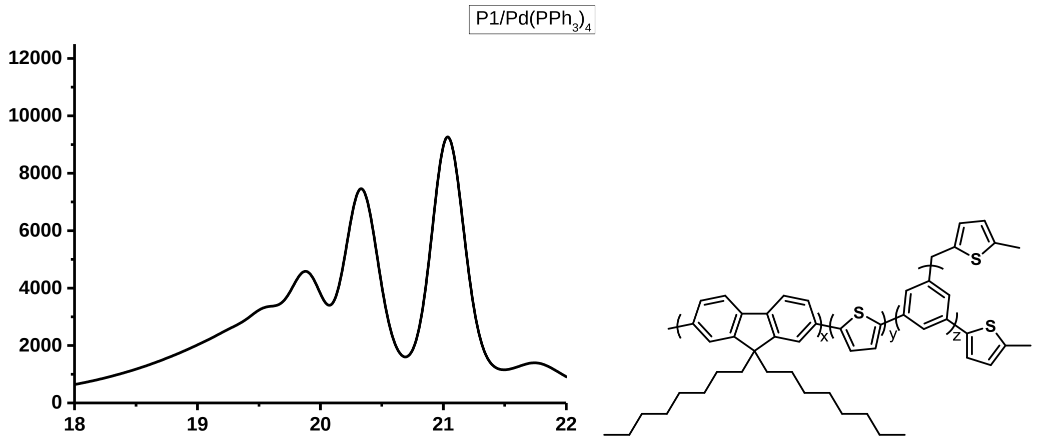

Figure S2. GPC elution curve of polymer **P1** prepared by using $Pd(PPh_3)_4$ as the catalyst precursor. Molecular weight determined by GPC with THF as eluent, calibrated with polystyrene standards.

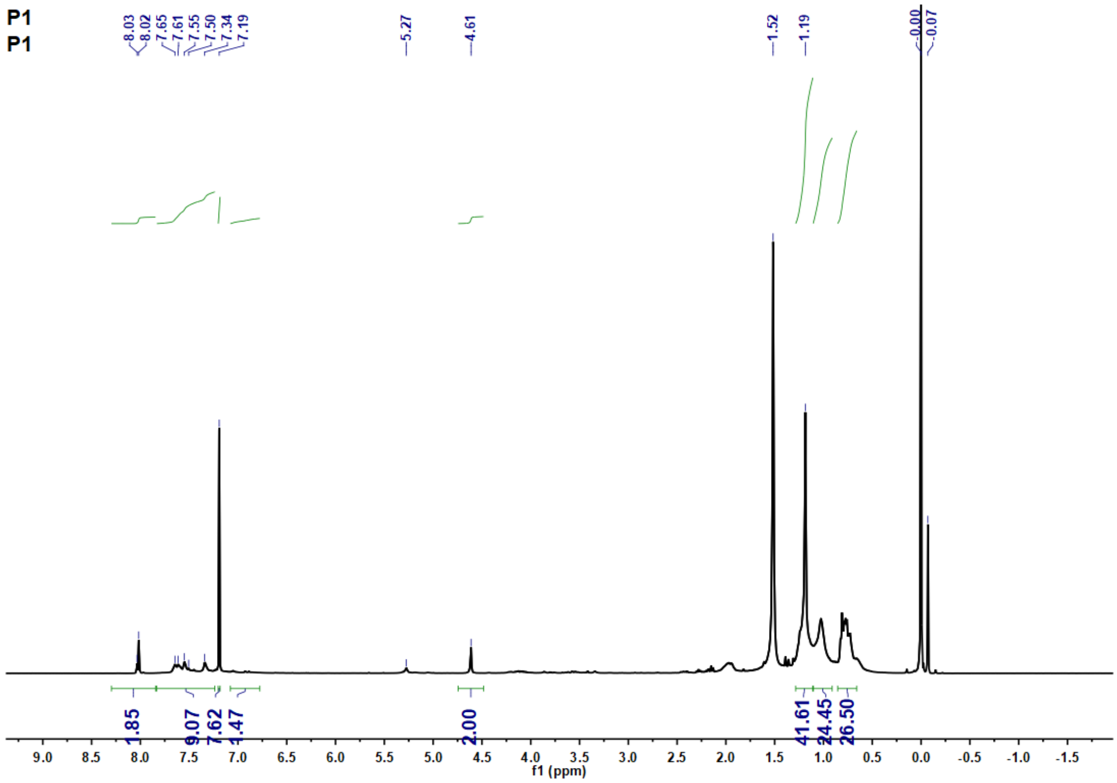

Figure S3. ^{1}H NMR spectrum of polymer

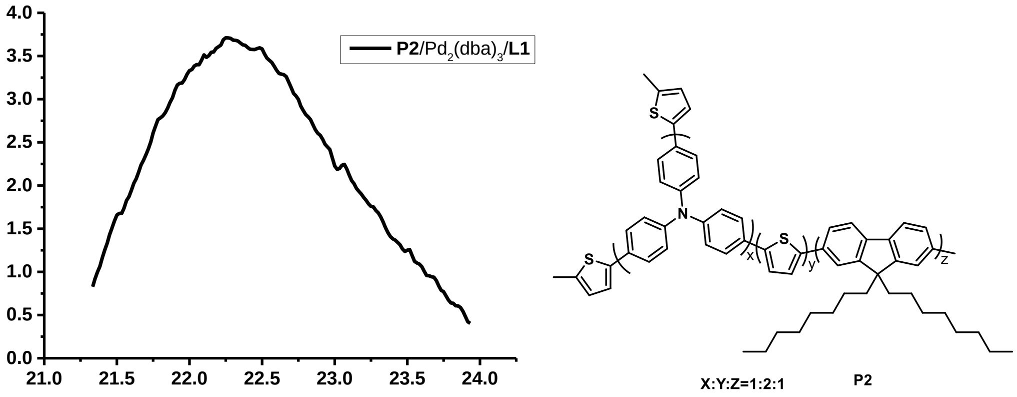

Figure S4. GPC elution curve of polymer **P2** prepared by using Pd2(dba)3/**L1**as the catalyst precursors. Molecular weight determined by GPC at 150 °C with 1,2,4-trichlorobenzene as eluent, calibrated with polystyrene standards.

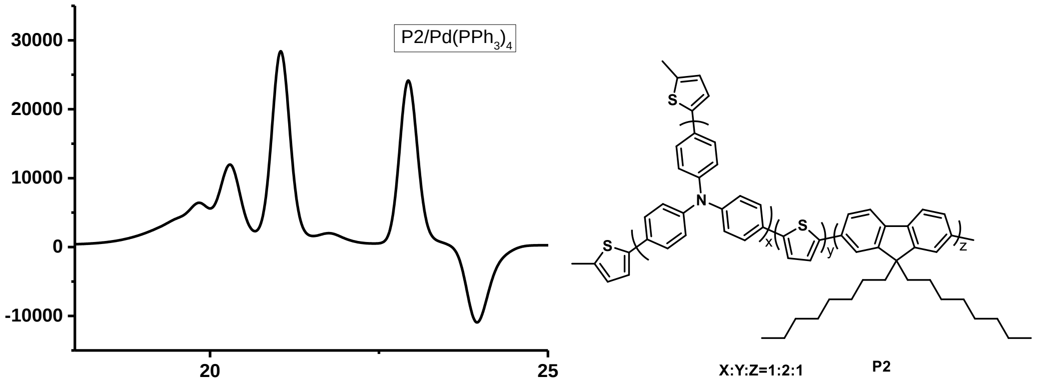

Figure S5. GPC elution curve of polymer **P2** prepared by using $Pd(PPh_3)_4$ as the catalyst precursor.

Molecular weight determined by GPC with THF as eluent, calibrated with polystyrene standards.

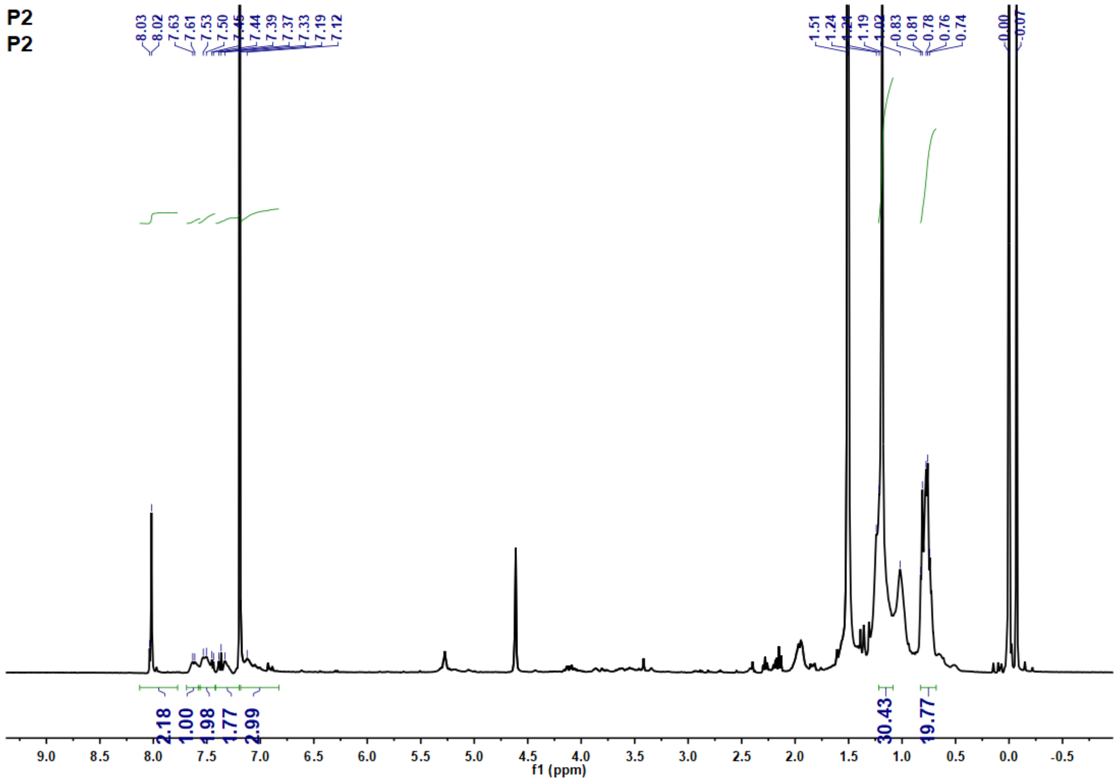

Figure S6. ^{1}H NMR spectrum of polymer **P2.**

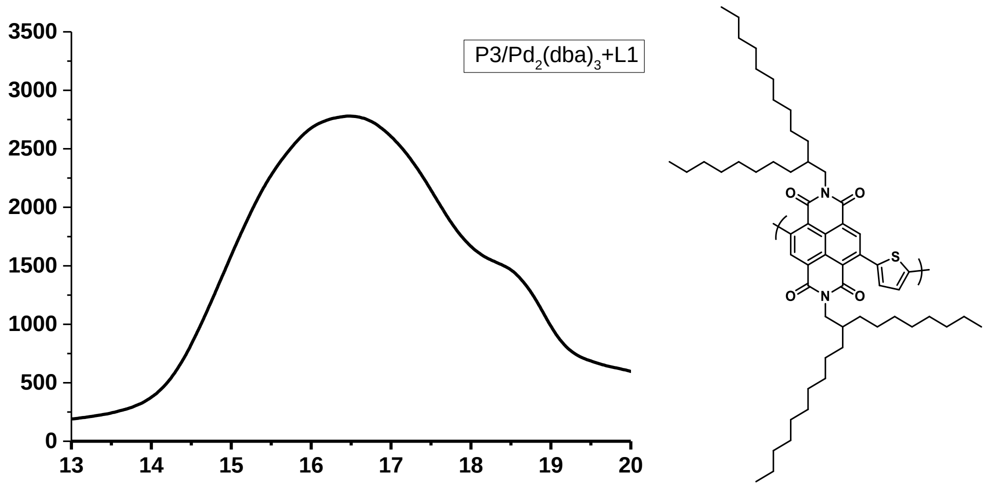

Figure S7. GPC elution curve of polymer **P3** prepared by using $Pd_2(dba)_3$/**L1** as the catalyst precursors. Molecular weight determined by GPC with THF as eluent, calibrated with polystyrene standards.

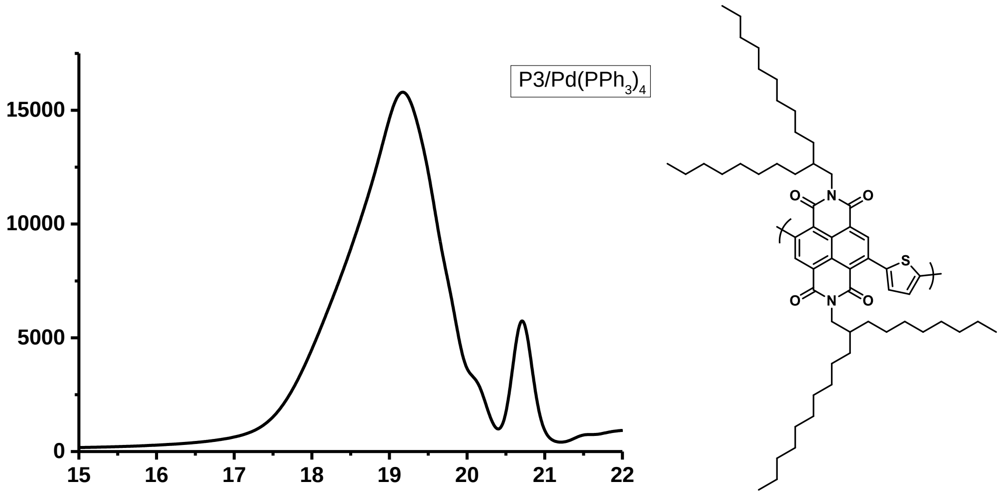

Figure S8. GPC elution curve of polymer **P3** prepared by using $Pd(PPh_3)_4$ as the catalyst precursor. Molecular weight determined by GPC with THF as eluent, calibrated with polystyrene standards.

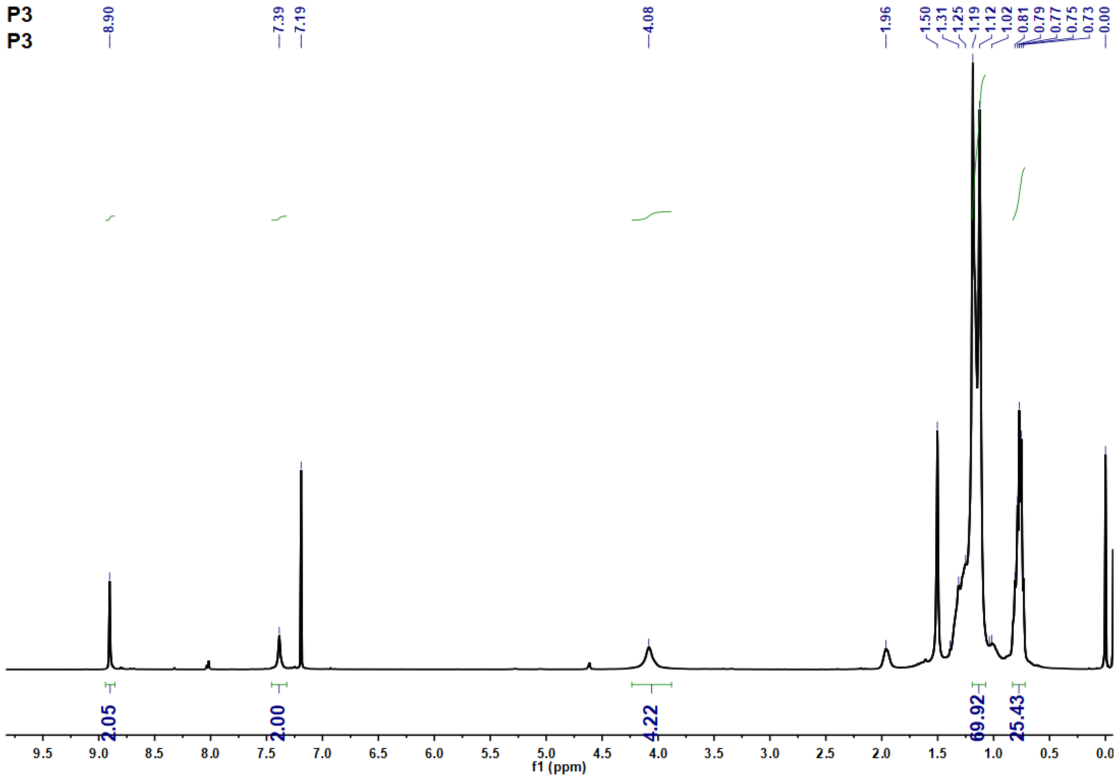

Figure S9. ^{1}H NMR spectrum of polymer **P3.**
